# Factors of Importance for Continuing Education After Primary School in Young People With Neuromuscular Diseases—Patient-Reported Outcomes From a National Survey

**DOI:** 10.1155/ijpe/2604359

**Published:** 2025-05-30

**Authors:** Charlotte Handberg, Kristin Allergodt, Annette Mahoney, Ann-Lisbeth Højberg

**Affiliations:** ^1^Research and Development, National Rehabilitation Center for Neuromuscular Diseases, Aarhus, Denmark; ^2^Department of Public Health, Faculty of Health, Aarhus University, Aarhus, Denmark

**Keywords:** adolescent, children, disability, education, neuromuscular disorders, rehabilitation, support

## Abstract

**Rationale:** Young people with neuromuscular diseases (NMDs) are especially at risk of being absent from school because of various symptoms, consequences of their disease, and frequent hospital visits. Growing up with a chronic disease can entail an increased risk of poor educational outcomes.

**Aims:** The study is aimed to investigate factors of importance for continuing with upper secondary and/or higher education after primary education when living with NMD, including expectations, support, and accessibility. In addition, we wanted to assess educational absence, discontinuation of studies, motivation, and sense of belonging.

**Method:** This cross-sectional study was founded in a national online questionnaire survey based on patient-reported outcomes from people with NMD. Five hundred and one persons were invited to participate. Data were analyzed using IBM SPSS Statistics 26. All variables were presented as numbers and percentages.

**Results:** Responses were obtained from 172 (34.3%) young people with NMD. Twenty (11.6%) answered that their parents'/relatives' educational expectations for them were lower than their own expectations. Forty (24.4%) answered that their teachers seldom or never planned the lessons in ways that were inclusive for them. Forty-five (27.7%) responded that they were always or mostly more absent than their classmates in primary and lower secondary education. Thirty-two (24.8%) respondents who had started upper secondary education had dropped out of one or more educational programs. Thirty-one (18.6%) answered they seldom or never had a sense of belonging at school.

**Conclusion:** Our results show novel knowledge on factors of importance for continuing education after primary school in young people with NMD. We found that teachers and parents carry a responsibility to show expectations to young people with NMD to ensure that the young people acquire adequate academic skills and actively participate in classroom activities. Supportive initiatives to prevent loneliness are important for keeping young people with NMD in the educational system.

## 1. Introduction

The term neuromuscular disease (NMD) covers a range of rare hereditary diseases, all progressive and sometimes with poor prognosis [1–3]. Moreover, the available treatment options for most of the diseases are limited or nonexisting [1–3]. The diseases are distinguished by a progressive loss of muscular strength, muscular atrophy, fatigue, pain, bulbar symptoms, and negatively affected quality of life [1–3]. Early life determinants of health for this group of people involve physical, emotional, practical, and social difficulties, which may affect their health and social position negatively [4, 5]. Young people with NMD are at risk of being absent from school and leisure activities because of impaired functioning, various symptoms, and consequences of the NMD and frequent hospital evaluations and follow-ups [6].

The Danish educational system is financed for the larger proportion (around 92%) by the public welfare system [7]. Education is compulsory from 0 to 9 grade, with an optional 10th grade [8]. Upper secondary education includes general upper secondary education and vocational education and training. Students qualify for higher education with their grades from upper secondary education or through admission tests. Young people generally start Grade 0 the year they turn six [8].

In general, people with disabilities attain lower education and manage poorer in life [9]. Research has shown that educational absence due to chronic disease can lead to educational and social setbacks and enforce educational disengagement [10–12], and growing up with chronic disease can entail an increased risk of poor educational outcomes [11]. People with NMDs are known to have a more dispersed pattern of absence than the background population, with around 2–4 days of absence per month during their entire educational span and often without any possibility of receiving compensatory education [13]. Most people with NMD live well into adulthood, and thanks to new and improved treatment methods, this also applies to diagnoses that were traditionally associated with short life expectancy, such as Duchenne muscular dystrophy (DMD) where median life expectancy has risen to approx. 30 years [14–16]. Therefore, it is fundamental to ensure that young people with NMD get an education to enhance their quality of life and independence. The increased risk of absence from education is unfortunate for young people with NMD since education is a strong predictor of how well they do in life in regard to health, income, employment, social inclusion, and working life conditions [17, 18]. The aim of this study is therefore to investigate factors of importance for continuing with upper secondary and/or higher education after primary education when living with NMDs, including expectations, support, and accessibility. In addition, we wanted to assess educational absence, discontinuation of studies, motivation, and sense of belonging.

## 2. Methods

### 2.1. Design

This cross-sectional study was founded on a national online questionnaire survey based on patient-reported outcomes from young people with NMD.

### 2.2. Participants and Procedure

In January 2023, the online questionnaire survey was sent to 501 people (≥ 18–30 years of age) with NMD and registered at the National Rehabilitation Center for Neuromuscular Diseases (RCFM) [19, 20]. The online questionnaire was developed in SurveyXact (Ramboll Denmark). Questions in the survey were developed based on two semistructured focus group interviews [21]. Furthermore, the questions for the survey were inspired by clinical knowledge from the professionals at RCFM, exciting literature, and various surveys on the background population in Denmark in the same age group. The link to the survey was sent through a secure digital mailbox (*n* = 494). Those who did not have a digital mailbox received a link to the survey by email (*n* = 7). A letter accompanying the link to the survey questionnaire contained information about the project and stated that answering the survey would be counted as consent. People were asked to complete the survey regardless of their educational or employment status to reduce response bias. A reminder was sent to everyone after 1 week and again after 5 weeks. Two hundred and eight young people answered the survey, resulting in a response rate of 41.5%.

### 2.3. The Structure of the Questionnaire

The questionnaire consisted of questions that all respondents had to answer (in the following referred to as “all”) in addition to questions that respondents were routed to based on their previous answers, for example, questions related to higher education (bachelor's and master's program) that were only accessible for those who answered that they had attended or were attending higher education. This resulted in the following structure: (a) *demographic information* (all), (b) *primary and lower secondary education* (*0*–*9 grade*) (all), (c) *upper secondary education* (*10–12 grade*) (all attending or having attended one or several upper secondary education(s)), (d) *higher education* (all attending or having attended one or more higher education), (e) *only primary and lower secondary education* (*0–9 grade*) (all who had not moved on to upper secondary), and (f) *concluding questions* (all).

Participants attending or having attended higher education were asked to relate to statements about upper secondary school as well as higher education.

### 2.4. Questions in the Questionnaire and Patient-Reported Outcomes

• *Demographic information*: age, sex, diagnosis, primary and lower secondary education, enrollment in upper secondary education, the highest level of completed education, current employment, ambulation, and other functional levels (difficulties in writing or focusing, lack of concentration, and need for breaks).
• *Expectations, support, and reactions*: self-efficacy, expectations from parents, expectations from teachers, expectations from others and self, support from others (teachers, fellow students, student counsellor, parents, or siblings), social activities, sympathy from others, motivation, and one's own and others' reactions to NMD.
• *Accessibility and educational support*: assistive devices, personal assistance, mentoring, and exemptions.
• *Absence*: frequency of and reasons for school absence (illness, fatigue, pain, demands, treatment, money, and personal or practical help).
• *Discontinuation of studies*: reasons for dropping out (expectations not met, money, time, demands, dreams, and wishes).

### 2.5. Patient and Public Involvement

The project protocol and survey were qualified continuously by the author group in collaboration with an advisory group of people with NMD (*n* = 4) and professionals from clinical practice at RCFM (*n* = 6).

### 2.6. Statistical Methods

Data were analyzed using IBM SPSS Statistics 26. Participants who had only answered questions on demographics were excluded from the data analysis. All categorical variables were presented as numbers and percentages. Categories in which respondents could mark multiple answers were presented as numbers.

### 2.7. Ethics

The study was conducted in accordance with the Helsinki Declaration of 1975 [22]. According to the Central Denmark Region Committees on Biomedical Research Ethics, the project was not liable to notification (Request No. 68/2022, Jr.no. 1-10-72-1-22). According to the Consolidation Act on Research Ethics Review of Health Research Projects, Consolidation Act Number 1338 of 1 September 2020, Section 14 (2), notification of questionnaire surveys or medical database research projects to the research ethics committee system is only required if the project involves human biological material. Therefore, this study could be conducted without approval from the committees.

## 3. Results

### 3.1. Study Population

Participant characteristics are presented in Table 1. Five hundred and one people with NMD received the questionnaire and 208 answered. Thirty-six had only answered questions on demographics and were excluded from the analysis. One hundred seventy-two (34.3%) people with NMD were included in the study; 136 people completed the questionnaire and 36 completed it partially. The people included were between 18 and 30 years old, with the majority being between 21 and 25 (*n* = 70, 40.7%). An equal number of women and men answered the questionnaire. Over 20 diagnoses were represented in the survey. The most common diagnoses represented were DMD (*n* = 26, 15.1%), Charcot-Marie-Tooth (CMT) (*n* = 22, 12.8%), myotonic dystrophy type 1 (DM1) (*n* = 16, 9.3%), and spinal muscular atrophy (SMA) types 2 and 3 (*n* = 15, 8.7%). One hundred thirty-seven (81.5%) respondents had attended ordinary primary and lower secondary school (0–9 grade), while 31 (18.5%) had attended a special needs class or other type of school (0–9 grade). One hundred forty-four (89.4%) respondents were or had been enrolled in upper secondary education (10–12 grade), while 17 (10.6%) respondents had not been enrolled in upper secondary education. The highest level of education completed by most respondents was upper secondary education (*n* = 60, 56.6%), followed by a bachelor program or academy profession program (*n* = 29, 27.4%). One hundred seven (62.2%) respondents could walk, while 65 (37.8%) used a wheelchair. Fifty-nine (34.3%) respondents had always or mostly had difficulties in writing for long periods of time. Twenty-eight (16.3%) respondents had always or mostly had difficulties in focusing on schoolwork or other tasks. Fifty-three (30.8%) respondents always or mostly needed many breaks during the day to keep up their energy.

### 3.2. Expectations, Support, and Reactions

Table 2 refers to results about expectations, support, and reactions from surroundings.

All participants were asked about educational expectations from parents/relatives and teachers. Twenty (11.6%) answered that their parents'/relatives' educational expectations for them were lower than their own expectations. Nineteen (11.1%) answered that their parents'/relatives' educational expectations were lower for them than for their siblings or other people at their age. Furthermore, 24 (14.0%) answered that their teachers' educational expectations were lower for them than for their classmates.

Respondents who were enrolled or had been enrolled in upper secondary education were asked about valuable support and lack of support during upper secondary education. They could choose between multiple answers. We identified 178 answers regarding lack of support. Respondents mostly lacked support from teachers (*n* = 27), student counsellors (*n* = 20), and fellow students (*n* = 16).

All participants were asked to consider statements about sympathy and reactions from other people. Responses were obtained from 17 respondents with only primary and lower secondary education, 138 with upper secondary education, and 45 with higher education. Six (35.2%) of those with only primary and lower secondary education answered that people had always or most of the time been overly considerate of their NMD. In contrast, 17 (12.3%) with upper secondary education and 15 (11.1%) with higher education answered the same.

### 3.3. Accessibility and Educational Support

Table 3 shows results about accessibility and educational support.

All participants were asked to answer questions about educational and physical accessibility in primary and lower secondary school (0–9 grade). One hundred and sixty-four responded to these questions. Forty (24.4%) respondents answered that their teachers seldom or never planned the lessons in ways that were inclusive for them. Additionally, 32 (19.5%) answered that they seldom or never had access to assistive devices and personal assistance when they needed it.

### 3.4. Frequency of and Reasons for School Absence

Table 4 presents results on the frequency of and reasons for school absence. All participants were asked about absence in school; 162 responded to questions about absence in primary and lower secondary education, 130 responded to questions about absence in upper secondary education, and 45 responded to questions about absence in higher education. Forty-five (27.7%) responded that they were always or mostly more absent than their classmates in primary and lower secondary education. Thirty-seven (28.4%) responded that they were always or mostly more absent than their classmates in upper secondary education. In contrast, the figure for higher education was only 8 (17.8%). Those who answered that they were always, mostly, or sometimes more absent than their classmates were asked to elaborate on the reasons. The most common reasons for having more absence were that they were more tired (*n* = 47/45/12), more often ill (*n* = 36/31/6), often in pain (*n* = 26/22/5), or spent more time on treatment (*n* = 54/29/7) compared to their peers.

### 3.5. Discontinuation of Studies

Table 5 presents results on the discontinuation of studies and reasons for not completing education.

The participants who were or had been enrolled in upper secondary and higher education were asked about discontinuation of studies; 129 responded to questions about upper secondary education and 45 to questions about higher education. Thirty-two (24.8%) respondents who had started upper secondary education had dropped out of one or more educational programs. Thirteen (28.9%) respondents who had started higher education had done the same.

Seventeen participants who had only attended primary and lower secondary education were asked about reasons for not having moved on to upper secondary education. The most common reasons were that they did not have the energy (*n* = 10), had not found themselves smart enough (*n* = 7), or did not feel like it (*n* = 6).

### 3.6. Motivation and Sense of Belonging

Table 6 presents results on motivation and sense of belonging.

All participants were asked about their motivation in primary and lower secondary school. One hundred sixty-seven responded to the questions. Thirty-one (18.6%) answered they seldom or never had a sense of belonging at school. Thirty-seven (22.2%) answered that it was always or mostly difficult to participate in social activities.

Furthermore, all participants were asked to consider statements about their motivation; responses were obtained from 17 participants with only primary and lower secondary education, 141 participants with upper secondary education, and 46 with higher education. Many respondents answered that they always or mostly felt bad if they could not overcome the same things as their peers: 7 (41.1%) with only primary and lower secondary education, 39 (27.6%) with upper secondary education, and 14 (30.4%) with higher education, respectively.

All participants were asked to answer statements about their social life: 17 participants with only primary and lower secondary education, 130 with upper secondary education, and 45 with higher education answered the statements. Six (35.2%) with only primary and lower secondary education answered that they had always or mostly given social activities low priority to save energy for their education/a job or other activities. This was a high percentage compared to those with upper secondary education (25, 19.3%) and higher education (8, 17.8%).

## 4. Discussion

To our knowledge, this is the first study to investigate factors of importance for continuing with upper secondary and/or higher education after primary education for people with NMDs between 18 and 30 years of age. Our findings represent over 20 different neuromuscular diagnoses.

### 4.1. Study Population

The most common diagnoses represented in our study were DMD with 15.1%, CMT with 12.8%, and DM1 with 9.3%. The large representation of people with DMD in our study was surprising, since it is one of the smaller diagnosis groups registered with RCFM with around 4% [19]. Diagnoses like DM1 and DMD carry a risk of cognitive deficits and intellectual impairment, and the percentage of participants with this specific diagnosis was well represented in our study [23–26].

Out of all the respondents, 81.5% had attended ordinary primary and lower secondary school, while 18.5% had attended a special needs class. When looking at the background population in Denmark for the years 2022/2023, only 5.1% attended a special needs class [27]. There may be various explanations for the relatively high number of young people with NMD in a special needs class or other type of school. Around 34% had always or mostly had difficulties in writing for longer periods of time, and 31% stated that they always or mostly needed many breaks during the day to keep up their energy. Literature shows that people with disabilities are more likely to be less educated due to challenges related to an impaired function [16, 28]. Our findings showed that 89.4% of the participants were or had been enrolled in upper secondary school, and the highest level of education completed by most respondents was upper secondary education (56.6%), followed by a bachelor program or academy profession program (27.4%). The numbers for enrolling in upper secondary school in this study are lower than for the background population in which 91.6% applied for upper secondary education or vocational education in 2022 [29]. These findings are important since research has shown that educated people with disabilities seem to cope and function better with their disabilities in general [30]. Furthermore, education is known to be of importance for how well you do in life in regard to health, income, employment, social inclusion, and working life conditions [17, 18].

### 4.2. Expectations, Support, and Reactions

Some respondents found that their parents' expectations for their education did not match their own (11.6%) or those for their siblings (11.1%) and that their teachers had lower expectations for them than for their classmates (14.0%). This has also been found in prior research showing how teachers of students with DMD tended to have lower expectations of these students compared to their peers [31]. Another study illustrated how only 63% of the teachers in schools without special needs offers and none of the teachers in schools with special needs offers expected their students with a NMD to graduate [31]. This is unfortunate since research has shown a link between expectations and academic achievement and that teachers' expectations are of great importance for students' achievement [32]. Low expectations can have negative effects which can accumulate over time, adding up to significant differences in educational outcomes [32]. In a recent qualitative study on perspectives of young people with NMDs regarding their educational choices, the participants emphasized the importance of expectations of their parents and teachers and explained the expectations as helpful [33]. The participants all underscored the positive influence expectations had on making choices for themselves, their education, and a future work life [33]. The low expectations experienced by the participants in the present study might be related to overprotection of the child or student with NMD, but knowing that low expectations are harmful, the intention to protect may have the opposite effect. Therefore, parents and teachers must be made aware of the false assumption that young people with disabilities cannot achieve the same as their peers or be successful in school.

### 4.3. Accessibility and Educational Support

Of the respondents, 24.4% answered that their teachers seldom or never planned the lessons in ways that were inclusive for them and 19.5% that they seldom or never had access to assistive devices and personal assistance when they needed it. Learning disabilities among people with certain NMD diagnoses such as DMD and DM1 have been shown to affect academic skills and personal relations negatively [34]. In Denmark, education for students with special needs should, if possible, be implemented in mainstream schools, and all young people are entitled to education adapted to their prerequisites and needs [35]. Feeling academically and socially behind can make it difficult to catch up and entail feelings of being excluded and alone. Being excluded can affect quality of life negatively, and therefore, it is important to intervene with supportive and inclusive measures in class and ensure that assistive devices and personal assistance are available when needed [34]. Teachers carry a responsibility to communicate with the young people with NMD and their parents and to ensure that the young people acquire adequate academic skills and that they can actively participate in classroom activities [36].

### 4.4. Absence and Discontinuation of Studies

Around 28% of all the respondents answered that they were mostly or always more absent than their classmates in primary and lower secondary school. Of the respondents, 28.4% who were or had been enrolled in upper secondary education answered that they were always or mostly more absent than their classmates. When looking at the background population, the average absence for a child in primary school was 8% in the school year 2021/2022 [8]. The most common reasons for being absent mentioned by the respondents in our study were that they were tired, ill, in pain, or getting treatment. A recent study has shown differences in the registration of school absence and requirements for attendance in Scandinavia, emphasizing the need to focus on opportunities for educational support for young people with disabilities and school absence [37].

Education and schooling have been shown to improve health and reduce adult mortality, making the importance of increased investment in education essential [38]. From our results, we do not know why absence is lower among respondents enrolled in higher education, but they may experience fewer symptoms from their disease and, thus, be more likely to continue in the educational system. Therefore, it is essential that schools and educational programs are inclusive and flexible to ensure that all students with NMD can attend school and continue in the educational system even if they experience symptoms like illness, fatigue, or pain [36]. Technologies like online education or telepresence robots may be means to secure adherence to education for those at risk of frequent or prolonged absences [13, 39].

### 4.5. Motivation and Sense of Belonging

Many respondents answered that they always or mostly felt bad if they could not overcome the same things as their peers; this was more common for those with only primary and lower secondary education. Asked about belonging at primary and lower secondary school, 18.6% of all participants answered that they seldom or never had felt a sense of belonging and 22% that it was always or mostly difficult to participate in social activities. This is unfortunate since it is important for people to feel valued and to belong to a social group as this creates a dimension of social identity [39, 40]. Our results showed that around 35% of the people with only primary and lower secondary education answered always or mostly to have given social activities lower priority to save energy for their education, a job, or other activities. This might indicate that people with low educational status are less prone to engage in social communities and, thus, at risk of becoming more isolated and lonelier. Therefore, it is important to ensure that students with NMD receive help and support to cope with the symptoms of their disease in order to strengthen participation in school and academic activities all the way through the educational system. For future research, it is relevant to investigate whether students with certain diagnoses are more exposed to educational absence and/or drop out of their education. Moreover, it would be relevant to investigate which paths those who drop out of the educational system choose in life.

### 4.6. Strength and Limitations

Out of 501 invited participants, 172 people with NMD were included in the analysis, resulting in a response rate of 34.3% which is considered good in a survey. Our sample represents participants of various ages within the inclusion criteria and a broad spectrum of NMD diagnoses vouching for the transferability to other contexts, countries, and populations of young people with disability. Unfortunately, we do not know who the nonresponders were and whether they differ from the respondents in terms of age, sex, educational level, or function. Furthermore, the sample of our study population with only primary and lower secondary education was small with only 17 respondents (out of the total sample of 172), making it difficult to draw strong conclusions based on this group.

The study is based on a patient-reported questionnaire in which participants were asked to answer questions about their schooling. For some respondents, recall bias might have affected their answers since it can be difficult to remember exactly how they experienced their schooling after several years. Additionally, the questions about schooling alternated between general and more specific educational questions, which might have been difficult to navigate. Finally, some of the participants were not diagnosed with NMD until after they had finished school, and this might also have affected their answers. Nevertheless, our results present novel and important information on factors of importance for continuing education after primary school in young people with NMDs.

## 5. Conclusion

Our results show novel knowledge on factors of importance for continuing education after primary school in young people with NMDs. We found that a higher percentage of young people with NMD were attending special needs classes than the background population, special needs that may be related to cognition, functioning, and/or fatigue. We also found that teachers carry a responsibility to communicate with young people with NMDs and their parents and to ensure that the young people acquire adequate academic skills and that they can actively participate in classroom activities. Supportive initiatives to prevent loneliness, including social skills training, increased social support, opportunities for social interaction, and cognitive behavioral exercises, are important for keeping young people with NMD in the educational system. It is essential to ensure inclusion in education by informing about options for help and assistance, including the possibility of prolonging studies if needed. Additionally, it is important to ensure accessible education adapted to specific needs to support young people with NMDs and school absence. It is important to integrate technological solutions such as online lessons or telepresence robots to ensure education for students hindered by illness or fatigue. Finally, parents and teachers must be made aware of the importance of expressing expectations to young people with NMDs to influence their choices regarding education and future work life positively. We believe that our findings can be transferred to other groups of people with disabilities, contexts, countries, and chronic diseases.

## Figures and Tables

**Table 1:** Characteristics of the respondents.

				*n* (%)		

Age (*n* = 172)						
18–20				40 (23.3)		
21–25				70 (40.7)		
26–30				62 (36.0)		
Sex (*n* = 172)						
Woman				86 (50.0)		
Man				86 (50.0)		
Diagnosis (*n* = 172)						
Duchenne muscular dystrophy (DMD)				26 (15.1)		
Charcot-Marie-Tooth (CMT)				22 (12.8)		
Myotonic dystrophy type 1 (DM1)				16 (9.3)		
Spinal muscular atrophy (SMA) type 2 + 3				15 (8.7)		
Limb–girdle muscular dystrophy				13 (7.6)		
Facioscapulohumeral muscular dystrophy (FSHD)				12 (7.0)		
Congenital muscular dystrophy				10 (5.8)		
Myasthenia gravis (MG)				9 (5.2)		
Becker muscular dystrophy				6 (3.5)		
Congenital myopathy				6 (3.5)		
Other neuromuscular diseases (NMD)^a^				34 (19.8)		
Not stated				3 (1.7)		
Primary and lower secondary education (0–9 grade) (*n* = 168)						
Ordinary primary and lover secondary school				137 (81.5)		
Special needs class/other type of school				31 (18.5)		
Enrollment in upper secondary education (10–12 grade) (*n* = 161)						
Yes				144 (89.4)		
No				17 (10.6)		
Highest level of completed education (*n* = 106)						
Upper secondary education (10–12 grade (academic, vocational, or other upper secondary education))				60 (56.6)		
Bachelor program or academy profession program				29 (27.4)		
Master's program				9 (8.5)		
Other adult education				8 (7.5)		
Current employment (*n* = 138)						
Paid work (full time job, part-time job, job on special terms)				36 (26.1)		
Exploring job opportunities (e.g., in a temporary job to test the ability to work)				15 (10.9)		
Disability pension/job with salary subsidy to employer for people with disability pension				29 (21.0)		
Enrolled in a further education program (academic or profession)				35 (25.4)		
Other				23 (16.7)		
Ambulation (*n* = 172)						
Can walk unaided (no use of assistive device or help from another person)				81 (47.1)		
Can walk but sometimes need help from a person or from an assistive device				26 (15.1)		
Use a wheelchair and can manage most things without assistance				4 (2.3)		
Use a wheelchair and sometimes or mostly need personal assistance				61 (35.5)		

Other functional level (*n* = 172)						
	**Always**	**Mostly**	**Sometimes**	**Seldom**	**Never**	**Do not know**

Difficulties in writing for long periods of time	28 (16.3)	31 (18.0)	39 (22.7)	37 (21.5)	33 (19.2)	4 (2.3)
Difficulties in focusing on schoolwork or other tasks	7 (4.1)	21 (12.2)	75 (43.6)	49 (28.5)	20 (11.6)	0 (0.0)
Need many breaks during the day to keep up energy	19 (11.0)	34 (19.8)	66 (38.4)	30 (17.4)	20 (11.6)	3 (1.7)

^a^Other NMDs represents diagnoses with < 6 respondents (e.g., collagen 6, Duchenne carrier, myotonic dystrophy type 2, Emery–Dreifuss muscular dystrophy, mitochondrial myopathy, myotonia congenita, paramyotonia, periodic paralysis, Pompe disease) and those answering “other” in the questionnaire. In all, over 20 diagnoses were represented in the survey.

**Table 2:** Expectations, support, and reactions of young people with NMD.

Expectations from others and self						
*Which of the following descriptions fits your parents'/relatives' expectations to your education the best? *(*n* = 172)						

Participant category^b^					All	
They were the same as my own expectations					91 (52.9)	
They were higher than my own expectations					27 (15.7)	
They were lower than my own expectations					20 (11.6)	
I do not know what my parents'/relatives' expectations for me were					34 (19.8)	

*Which of the following descriptions fits your parents'/relatives' expectations to your education compared to that of your siblings (if any) or other people at your age? *(*n* = 171)						

Participant category^b^					All	
They were the same of me as they were of my siblings or other children my age					86 (50.3)	
They were higher of me than of my siblings or other children my age					20 (11.7)	
They were lower of me than of my siblings or other children my age					19 (11.1)	
I do not know what my parents'/relatives' expectations were of me compared to my siblings or other children my age					34 (19.9)	
I do not have any siblings					12 (7.0)	

*Which of the following descriptions fits your teachers' expectations to your education compared to that of your classmates? *(*n* = 171)						

Participant category^b^					All	
They were the same of me as of my classmates					86 (50.3)	
They were higher of me than of my classmates					16 (9.4)	
They were lower of me than of my classmates					24 (14.0)	
I do not know what my teachers' expectations to my education was compared to that of my classmates					45 (26.3)	

*I think it is realistic that I will be occupied by the things I dream of in the future *(*n* = 138)						

Participant category^b^					All	
Yes					98 (71.0)	
No					14 (10.1)	
Do not know/not relevant					26 (18.8)	

**Support from others (questions asked in section about upper secondary education (10–12 grade))**						
	**Who have you received valuable academic or social support from during your upper secondary education? (Mark as many as you want)**				**Who did you lack support from during your upper secondary education? (Mark as many as you want)**	

Teachers	67				27	
Fellow students	67				16	
Student counsellor	30				20	
Mentor	8				3	
Peers with a neuromuscular disorder	10				5	
Other friends	47				9	
Parents or siblings	91				6	
National Rehabilitation Center for Neuromuscular Diseases	12				6	
The Danish Neuromuscular Foundation	11				6	
No one	6				43	
Do not know	6				36	
Other	5				1	

**Sympathy and reactions from others**						
*Which of the following statements have been applicable to you during your education/in general? *(*n* = 17/138/45)						

Response category	Always	Mostly	Sometimes	Seldom	Never	Do not know
Participant category^b^	Low	Low	Low	Low	Low	Low
	Middle	Middle	Middle	Middle	Middle	Middle
	High	High	High	High	High	High
My neuromuscular disease has been visible to others	8 (47.1)	2 (11.8)	3 (17.6)	2 (11.8)	2 (11.8)	0 (0.0)
	57 (41.3)	9 (6.5)	21 (15.2)	25 (18.1)	23 (16.7)	3 (2.2)
	21 (46.7)	3 (6.7)	4 (8.9)	7 (15.6)	8 (17.8)	2 (4.4)
I have thought about how others react to my neuromuscular disease	4 (23.5)	4 (23.5)	4 (23.5)	1 (5.9)	3 (17.6)	1 (5.9)
	20 (14.5)	29 (21.0)	44 (31.9)	21 (15.2)	18 (13.0)	6 (4.3)
	6 (13.3)	10 (22.2)	11 (24.4)	9 (20.0)	7 (15.6)	2 (4.4)
I have been open about my neuromuscular disease	11 (64.7)	1 (5.9)	2 (11.8)	2 (11.8)	1 (5.9)	0 (0.0)
	65 (47.1)	31 (22.5)	19 (13.8)	9 (6.5)	6 (4.3)	8 (5.8)
	29 (64.4)	6 (13.3)	4 (8.9)	1 (2.2)	3 (6.7)	2 (4.4)
It has been hard for me to find the right time to tell others about my neuromuscular disease	4 (23.5)	4 (23.5)	2 (11.8)	4 (23.5)	1 (5.9)	2 (11.8)
	22 (15.9)	17 (12.3)	16 (11.6)	28 (20.3)	38 (27.5)	17 (12.3)
	5 (11.1)	5 (11.1)	9 (20.0)	6 (13.3)	18 (40.0)	2 (4.4)
My teachers have been sympathetic of my neuromuscular disease	—	—	—	—	—	—
	42 (30.4)	43 (31.2)	16 (11.6)	13 (9.4)	5 (3.6)	19 (13.8)
	12 (26.7)	15 (33.3)	3 (6.7)	2 (4.4)	1 (2.2)	12 (26.7)
My fellow students/friends have been sympathetic of my neuromuscular disease	11 (64.7)	4 (23.5)	2 (11.8)	0 (0.0)	0 (0.0)	0 (0.0)
	49 (35.5)	42 (30.4)	18 (13.0)	9 (6.5)	4 (2.9)	16 (11.6)
	18 (40.0)	17 (37.8)	3 (6.7)	1 (2.2)	1 (2.2)	5 (11.1)
People have been reluctant to talk about my neuromuscular disease	2 (11.8)	1 (5.9)	1 (5.9)	5 (29.4)	3 (17.6)	5 (29.4)
	5 (3.6)	12 (8.7)	22 (15.9)	38 (27.5)	35 (25.4)	26 (18.8)
	1 (2.2)	2 (4.4)	11 (24.4)	13 (28.9)	11 (24.4)	7 (15.6)
I have been bullied because of my neuromuscular disease^a^	3 (17.6)	1 (5.9)	1 (5.9)	2 (11.8)	10 (58.8)	0 (0.0)
	1 (0.7)	2 (1.5)	9 (6.6)	19 (13.9)	93 (67.9)	13 (9.5)
	0 (0.0)	0 (0.0)	1 (2.2)	3 (6.7)	38 (84.4)	3 (6.7)
People have been overly considerate of me because of my neuromuscular disease	3 (17.6)	3 (17.6)	6 (35.3)	1 (5.9)	4 (23.5)	0 (0.0)
	7 (5.1)	10 (7.2)	43 (31.2)	34 (24.6)	27 (19.6)	17 (12.3)
	3 (6.7)	2 (4.4)	7 (15.6)	13 (28.9)	16 (35.6)	4 (8.9)

^a^Only 137 respondents in the category “middle” (one missing).

^b^Participant category refers to which respondents answered the question. All means all respondents answered the question. Low refers to all who had not moved on to upper secondary education. Middle refers to all attending or having attended one or several upper secondary education(s), as well as respondents moving on to higher education. High refers to all attending or having attended one or more higher education(s).

**Table 3:** Accessibility and educational support, for example, assistive devices, personal assistance, and mentoring, in young people with NMD.

**Physical and educational accessibility**						
*Which of the following statements match your experience of educational and physical accessibility in primary and lower secondary school (0–9 grade)? *(*n* = 164)						

Response category	Always	Mostly	Sometimes	Seldom	Never	Do not know
Participant category^b^	All	All	All	All	All	All
My academic performance in school was good	41 (25.0)	60 (36.6)	39 (23.8)	19 (11.6)	5 (3.0)	0 (0.0)
My teachers planned the lessons in ways that were inclusive for me	36 (22.0)	41 (25.0)	20 (12.2)	17 (10.4)	23 (14.0)	27 (16.5)
I was able to get around in the classrooms on equal terms with my classmates	101 (61.6)	42 (25.6)	11 (6.7)	4 (2.4)	3 (1.8)	3 (1.8)
I was able to get around in the schoolyard on equal terms with my classmates	97 (59.1)	50 (30.5)	10 (6.1)	2 (1.2)	2 (1.2)	3 (1.8)
I had access to assistive devices and personal assistance whenever I needed it	57 (34.8)	31 (18.9)	9 (5.5)	11 (6.7)	21 (12.8)	35 (21.3)

*Which of the following statements match you experiences of physical and educational accessibility in upper secondary school and further education? *(*n* = 133/45)						

Response category	Always	Mostly	Sometimes	Seldom	Never	Do not know
Participant category^b^	Middle	Middle	Middle	Middle	Middle	Middle
	High	High	High	High	High	High
I can/could participate in lessons/academic activities at my school^a^	57 (42.5)	62 (46.3)	12 (9.0)	1 (0.7)	1 (0.7)	1 (0.7)
	22 (48.9)	17 (37.8)	3 (6.7)	0 (0.0)	1 (2.2)	2 (4.4)
I can/could participate in internships if they are/were part of my education	62 (46.6)	20 (15.0)	10 (7.5)	6 (4.5)	4 (3.0)	31 (23.3)
	28 (62.2)	8 (17.8)	1 (2.2)	0 (0.0)	3 (6.7)	5 (11.1)
I can/could manage a student job if I want/wanted one	17 (12.8)	11 (8.3)	8 (6.0)	17 (12.8)	54 (40.6)	26 (19.5)
	10 (22.2)	1 (2.2)	4 (8.9)	1 (2.2)	21 (46.7)	8 (17.8)
It was necessary to switch to another educational program or school because of poor accessibility	9 (6.8)	2 (1.5)	9 (6.8)	7 (5.3)	93 (69.9)	13 (9.8)
	2 (4.4)	1 (2.2)	3 (6.7)	0 (0.0)	34 (75.6)	5 (11.1)

**Educational support**						

Response category	Always	Mostly	Sometimes	Seldom	Never	Do not know
Participant category^b^	Middle	Middle	Middle	Middle	Middle	Middle
	High	High	High	High	High	High
I was given sufficient information about educational support for students with special needs at my school (*n* = 49/27)	15 (30.6)	11 (22.4)	3 (6.1)	2 (4.1)	8 (16.3)	10 (20.4)
	6 (22.2)	6 (22.2)	4 (14.8)	6 (22.2)	2 (7.4)	3 (11.1)
I have received sufficient practical and/or personal help at my school (*n* = 57/15)	26 (45.6)	20 (35.1)	6 (10.5)	1 (1.8)	2 (3.5)	2 (3.5)
	11 (73.3)	3 (20.0)	0 (0.0)	0 (0.0)	1 (6.7)	0 (0.0)

*Which of the following statements match the guidance and support you have received at your school? *(*n* = 49/27)						

Response category	Always	Mostly	Sometimes	Seldom	Never	Do not know
Participant category^b^	Middle	Middle	Middle	Middle	Middle	Middle
	High	High	High	High	High	High
I have been granted exemption from exams or taken exams on special conditions (e.g., additional time) if needed	23 (46.9)	11 (22.4)	2 (4.1)	0 (0.0)	2 (4.1)	11 (22.4)
	8 (29.6)	7 (25.9)	1 (3.7)	1 (3.7)	2 (7.4)	8 (29.6)
I have been able to prolong my studies if needed	13 (26.5)	5 (10.2)	2 (4.1)	0 (0.0)	5 (10.2)	24 (49.0)
	10 (37.0)	3 (11.1)	0 (0.0)	1 (3.7)	2 (7.4)	11 (40.7)
I have been in doubt about where to apply for special needs support and/or exemptions	7 (14.3)	4 (8.2)	8 (16.3)	8 (16.3)	12 (24.5)	10 (20.4)
	3 (11.1)	1 (3.7)	5 (18.5)	2 (7.4)	9 (33.3)	7 (25.9)

*Which of the following statements matches your experience? *(*n* = 52/17)						

Participant category^b^						Middle
						High
I have had the assistive devices I needed during my education						46 (88.5)
						12 (70.6)
I have not had the assistive devices I needed during my education						6 (11.5)
						5 (29.4)

^a^One hundred thirty-four respondents in the category “middle.”

^b^Participant category refers to which respondents answered the question. All means all respondents answered the question. Middle refers to all attending or having attended one or several upper secondary education(s), as well as respondents moving on to higher education. High refers to all attending or having attended one or more higher education(s).

**Table 4:** Frequency of and reasons for school absence in young people with NMD.

**Absence**						

Response category	Always	Mostly	Sometimes	Seldom	Never	Do not know
Participant category^a^	All Middle High	All Middle High	All Middle High	All Middle High	All Middle High	All Middle High
Have been more absent than classmates/fellow students (*n* = 162/130/45)	20 (12.3) 18 (13.8) 3 (6.7)	25 (15.4) 19 (14.6) 5 (11.1)	35 (21.6) 24 (18.5) 7 (15.6)	26 (16.0) 30 (23.1) 12 (26.7)	45 (27.8) 35 (26.9) 13 (28.9)	11 (6.8) 4 (3.1) 5 (11.1)

**The following has affected absence from school (** **n** = 57/61/15**)**						
					All/middle/high	

Was more tired than peers					47/45/12	
Was ill more often than peers					36/31/6	
Often in pain					26/22/5	
Had to spend more time on treatment (e.g., physiotherapy and hospitalization) than peers					54/29/7	
Activities of daily living (e.g., eating, getting dressed, and going to the bathroom) took longer time than for peers					15/12/–	
Did not feel like participating in lessons + did not feel like participating in studies/I did not find pleasure in studies					24/19/7	
Did not have sufficient practical or personal assistance and/or assistive devices					7/7/4	
Financial situation was insufficient					–/3/3	
Studies had to be prolonged because of the neuromuscular disease					–/12/3	
Other reasons for absence					28/18/5	

^a^Participant category refers to which respondents answered the question. All means all respondents answered the question. Middle refers to all attending or having attended one or several upper secondary education(s), as well as respondents moving on to higher education. High refers to all attending or having attended one or more higher education(s).

**Table 5:** Discontinuation of studies and reasons for not completing education in young people with NMD.

**Discontinuation of studies, reasons for dropping out**		

Response category	Yes	No
Participant category^a^	Middle	Middle
	High	High
Have dropped out of one or more educational programs (*n* = 129/45)	32 (24.8)	97 (75.2)
	13 (28.9)	32 (71.1)

**Possible reasons for not going through with the studies (** **n** = 32/13**)**		

Participant category^a^		Middle/high
Discovered that the program was not as imagined		10/3
There were periods of illness or hospitalization related to the neuromuscular disease		10/3
Struggling to keep up with the academic level		13/4
Difficulties in participating in social activities at school		4/1
Difficulties in participating in social activities outside school		3/3
Did not have sufficient practical or personal assistance and/or assistive devices		1/0
Did not have enough money to cover the expenses of daily living during education		1/2
Did not have enough money to cover the expenses of daily living during prolonged education (e.g., was not granted additional funding from the State Education Fund) (further education)		–/1
Do not know the reason for dropping out		1/0
Have dropped out for other reasons		12/7

**Reasons for not starting upper secondary education after primary and lower secondary education (** **n** = 17**)**		

Participant category^a^		Low
Have not felt like it		6
Have not had the energy		10
Have not found oneself smart enough		7
Have never planned on getting an education		4
Needed time to figure out what want to do		3
The desired upper secondary schools were lacking in accessibility		2
Could not get the necessary support (e.g., assistive devices and personal help) to start upper secondary school		3
Did not have enough money to start school		2
Continuing with upper secondary education is not a tradition in the family		1
Have not started upper secondary education for other reasons		7

**Measures needed for going back to school (** **n** = 17**)**		

Participant category^a^		Low
More time for studies than peers		4
More practical and/or personal assistance and assistive devices than is available right now		1
Financial support		1
More guidance and information on options		4
None of the above		2

^a^Participant category refers to which respondents answered the question. Low refers to all who had not moved on to upper secondary education. Middle refers to all attending or having attended one or several upper secondary education(s), as well as respondents moving on to higher education. High refers to all attending or having attended one or more higher education(s).

**Table 6:** Motivation and sense of belonging in young people with NMD.

**Motivation and sense of belonging**						
*How do the following statements match your experience with primary and lower secondary school? *(*n* = 167)						

Response category	Always	Mostly	Sometimes	Seldom	Never	Do not know
Participant category^a^	All	All	All	All	All	All
I liked going to school	21 (12.6)	84 (50.3)	32 (19.2)	23 (13.8)	7 (4.2)	0 (0.0)
I had good friends in school	69 (41.3)	56 (33.5)	28 (16.8)	12 (7.2)	2 (1.2)	0 (0.0)
I was alone during recesses	4 (2.4)	14 (8.4)	33 (19.8)	54 (32.3)	62 (37.1)	0 (0.0)
I was bullied in school	11 (6.6)	8 (4.8)	22 (13.2)	36 (21.6)	87 (52.1)	3 (1.8)
I was accepted for who I was	65 (38.9)	56 (33.5)	20 (12.0)	13 (7.8)	9 (5.4)	4 (2.4)
I had a sense of belonging when I was at school	52 (31.1)	54 (32.3)	26 (15.6)	14 (8.4)	17 (10.2)	4 (2.4)
It was difficult for me to participate in social activities	13 (7.8)	24 (14.4)	62 (37.1)	38 (22.8)	29 (17.4)	1 (0.6)
I was not sure what I wanted to do after completing lower secondary education (9/10 grade)	30 (18.0)	22 (13.2)	28 (16.8)	22 (13.2)	60 (35.9)	5 (3.0)

*How do the following statements about motivation match your experience? *(*n* = 17/141/46)						

Response category	Always	Mostly	Sometimes	Seldom	Never	Do not know
Participant category^a^	Low	Low	Low	Low	Low	Low
	Middle	Middle	Middle	Middle	Middle	Middle
	High	High	High	High	High	High
I have felt different from my friends/fellow students	5 (29.5)	1 (5.9)	10 (58.8)	0 (0.0)	1 (5.9)	0 (0.0)
	17 (12.1)	14 (9.9)	50 (35.5)	34 (24.1)	26 (18.4)	0 (0.0)
	4 (8.7)	5 (10.9)	14 (30.4)	13 (28.3)	8 (17.4)	2 (4.3)
It is important for me to continue with upper secondary/further education	—	—	—	—	—	—
	96 (68.1)	22 (15.6)	23 (16.3)	8 (5.7)	3 (2.1)	0 (0.0)
	36 (78.3)	4 (8.7)	2 (4.3)	2 (4.3)	0 (0.0)	2 (4.3)
I am/have been happy with the schools/educational programs I attend/have attended	—	—	—	—	—	—
	54 (38.3)	53 (37.6)	23 (16.3)	8 (5.7)	3 (2.1)	0 (0.0)
	19 (41.3)	20 (43.5)	4 (8.7)	0 (0.0)	1 (2.2)	2 (4.3)
I have/had to push myself to keep up with my peers	—	—	—	—	—	—
	18 (12.8)	12 (8.5)	43 (30.5)	33 (23.4)	31 (22.0)	4 (2.8)
	6 (13.0)	9 (19.6)	9 (19.6)	13 (28.3)	7 (15.2)	2 (4.3)
I have felt bad if I could not overcome the same things as my peers	4 (23.5)	3 (17.6)	3 (17.6)	2 (11.8)	2 (11.8)	3 (17.6)
	14 (9.9)	25 (17.7)	25 (17.7)	28 (19.9)	37 (26.2)	12 (8.5)
	7 (15.2)	7 (15.2)	7 (15.2)	8 (17.4)	14 (30.4)	3 (6.5)
I have felt sad	5 (29.4)	2 (11.8)	6 (35.3)	2 (11.8)	1 (5.9)	1 (5.9)
	6 (4.3)	9 (6.4)	52 (36.9)	43 (30.5)	29 (20.6)	2 (1.4)
	2 (4.3)	4 (8.7)	15 (32.6)	13 (28.3)	10 (21.7)	2 (4.3)
I have been edgy and in a bad mood	4 (23.5)	2 (11.8)	7 (41.2)	3 (17.6)	1 (5.9)	0 (0.0)
	4 (2.8)	10 (7.1)	50 (35.5)	48 (34.0)	25 (17.7)	4 (2.8)
	2 (4.3)	2 (4.3)	12 (26.1)	19 (41.3)	9 (19.6)	2 (4.3)
I have been nervous	3 (17.6)	5 (29.4)	3 (17.6)	3 (17.6)	2 (11.8)	1 (5.9)
	9 (6.4)	17 (12.1)	55 (39.0)	32 (22.7)	27 (19.1)	1 (0.7)
	4 (8.7)	4 (8.7)	14 (30.4)	14 (30.4)	8 (17.4)	2 (4.3)
I have been able to talk to my parents if I was worried about something/I have had someone to talk to if I was worried about something	9 (52.9)	1 (5.9)	3 (17.6)	3 (17.6)	1 (5.9)	0 (0.0)
	64 (45.4)	39 (27.7)	16 (11.3)	9 (6.4)	8 (5.7)	5 (3.5)
	16 (34.8)	15 (32.6)	3 (6.5)	4 (8.7)	4 (8.7)	4 (8.7)
I have wanted to contribute to society	10 (58.8)	5 (29.4)	0 (0.0)	0 (0.0)	0 (0.0)	2 (11.8)
	—	—	—	—	—	—
	—	—	—	—	—	—
I have wanted to contribute to the school community	—	—	—	—	—	—
	52 (36.9)	49 (34.8)	29 (20.6)	3 (2.1)	4 (2.8)	4 (2.8)
	15 (32.6)	19 (41.3)	5 (10.9)	3 (6.5)	1 (2.2)	3 (6.5)
I have felt it was important to have something to wake up to	8 (47.1)	5 (29.4)	0 (0.0)	3 (17.6)	0 (0.0)	1 (5.9)
	—	—	—	—	—	—
	—	—	—	—	—	—

**Social life**						
*How do these statements about your social life match your experience? *(*n* = 17/130/45)						

Response category	Always	Mostly	Sometimes	Seldom	Never	Do not know
Participant category^a^	Low	Low	Low	Low	Low	Low
	Middle	Middle	Middle	Middle	Middle	Middle
	High	High	High	High	High	High
I have given social activities lower priority to have energy for my education/a job or other activities	3 (17.6)	3 (17.6)	4 (23.5)	1 (5.9)	4 (23.5)	2 (11.8)
	11 (8.5)	14 (10.8)	36 (27.7)	28 (21.5)	33 (25.4)	8 (6.2)
	4 (8.9)	4 (8.9)	15 (33.3)	9 (20.0)	11 (24.4)	2 (4.4)
I have missed out on social communities because of my neuromuscular disease	5 (29.4)	5 (29.4)	3 (17.6)	2 (11.8)	2 (11.8)	0 (0.0)
	5 (3.8)	13 (10.0)	40 (30.8)	25 (19.2)	36 (27.7)	11 (8.5)
	2 (4.4)	1 (2.2)	13 (28.9)	7 (15.6)	19 (42.2)	3 (6.7)
I have felt lonely	3 (17.6)	5 (29.4)	1 (5.9)	6 (35.3)	2 (11.8)	0 (0.0)
	7 (5.4)	12 (9.2)	42 (32.3)	39 (30.0)	28 (21.5)	2 (1.5)
	1 (2.2)	7 (15.6)	11 (24.4)	11 (24.4)	13 (28.9)	2 (4.4)
It was necessary that I took the initiative to meet with friends	4 (23.5)	6 (35.3)	4 (23.5)	0 (0.0)	2 (11.8)	1 (5.9)
	8 (6.2)	21 (16.2)	48 (36.9)	34 (26.2)	13 (10.0)	6 (4.6)
	1 (2.2)	5 (11.1)	13 (28.9)	14 (31.1)	7 (15.6)	5 (11.1)
I have arranged social gatherings to make sure I could participate	4 (23.5)	2 (11.8)	3 (17.6)	3 (17.6)	4 (23.5)	1 (5.9)
	2 (1.5)	11 (8.5)	18 (13.8)	33 (25.4)	60 (46.2)	6 (4.6)
	1 (2.2)	3 (6.7)	5 (11.1)	11 (24.4)	21 (46.7)	4 (8.9)
I have had the friends I wanted to have	7 (41.2)	5 (29.4)	4 (23.5)	0 (0.0)	0 (0.0)	1 (5.9)
	46 (35.4)	51 (39.2)	14 (10.8)	10 (7.7)	6 (4.6)	3 (2.3)
	20 (44.4)	11 (24.4)	6 (13.3)	4 (8.9)	2 (4.4)	2 (4.4)
I have had friends who also had a disability	5 (29.4)	1 (5.9)	3 (17.6)	3 (17.6)	3 (17.6)	2 (11.8)
	16 (12.3)	13 (10.0)	18 (13.8)	18 (13.8)	58 (44.6)	7 (5.4)
	5 (11.1)	5 (11.1)	3 (6.7)	9 (20.0)	21 (46.7)	2 (4.4)

*The personal assistant has sometimes been a barrier to friendships, social activities, or group work *(*n* = 14/58/15)						

Response category	Always	Mostly	Sometimes	Seldom	Never	Do not know
Participant category^a^	Low	Low	Low	Low	Low	Low
	Middle	Middle	Middle	Middle	Middle	Middle
	High	High	High	High	High	High
	1 (7.1)	3 (21.4)	4 (28.6)	1 (7.1)	5 (35.7)	0 (0.0)
	1 (1.7)	5 (8.6)	14 (24.1)	18 (31.0)	13 (22.4)	7 (12.1)
	1 (6.7)	0 (0.0)	3 (20.0)	7 (46.7)	3 (20.0)	1 (6.7)

^a^Participant category refers to which respondents answered the question. All means all respondents answered the question. Low refers to all who had not moved on to upper secondary education. Middle refers to all attending or having attended one or several upper secondary education(s), as well as respondents moving on to higher education. High refers to all attending or having attended one or more higher education(s).

## Data Availability

The data that support the findings of this study are available on request from the corresponding author. The data are not publicly available due to privacy or ethical restrictions.
